# Fe-Doped Barium Lanthanum Titanate as a Competitor to Other Lead-Free Piezoelectric Ceramics

**DOI:** 10.3390/ma15031089

**Published:** 2022-01-30

**Authors:** Beata Wodecka-Duś, Lucjan Kozielski, Jolanta Makowska, Mateusz Bara, Małgorzata Adamczyk-Habrajska

**Affiliations:** Faculty of Science and Technology, Institute of Materials Engineering, University of Silesia in Katowice, 41-500 Chorzów, Poland; beata.wodecka-dus@us.edu.pl (B.W.-D.); lucjan.kozielski@us.edu.pl (L.K.); jolanta.makowska@us.edu.pl (J.M.); mbara1@us.edu.pl (M.B.)

**Keywords:** BLT, lead-free multiferroic solid solution, piezoelectric coefficients

## Abstract

Multiferroic solid solutions of Ba_1−x_La_x_Ti_1−x/4_O_3_ and iron (BLFT) were synthesized using the conventional mixed oxide method. The dependence of the piezoelectric coefficients on Fe content in BLFT ceramics was determined by the quasi-static and resonance method. The results indicate that 0.3 mol% addition of Fe^3+^ ions to the ceramic structure increased the value of the piezoelectric parameter *d*_33_ to the maximum of 159 pC/N. This puts BLFT ceramics among other good-quality and lead-free piezoelectric ceramics. A major enhancement of dielectric properties related to the manipulation of Fe content in the barium lanthanum titanate (BLT) ceramics system is reported as well.

## 1. Introduction

The unique properties of ABO_3_ perovskite materials, so important from the point of view of modern electronics, are a consequence of a slight deformation of the regular crystal structure, which is maintained by isomorphic ion substitution and non-stoichiometry (as a result of the anionic and cationic vacancies formation). The A positions of the ions in the perovskite layer can be occupied by mono-, di-, and trivalent ions and rare earth cations, e.g., Na^+^, Ba^2+^, Ca^2+^, Sr^2+^, La^3+^, Bi^3+^ [1,2,3]. Smaller cations usually occupy the B positions, e.g., Fe^3+^, Ti^4+^, Zr^4+^, Ta^5+^, Nb^5+^, W^6+^ [4,5,6], characterized by a suitable size and oxidation [7,8]. The simultaneous substitution of admixtures in the A and B ion positions, in ABO_3_-type perovskites, is associated with obtaining original perovskite materials with complex electrical properties; examples include the compounds of (1−x)BiFeO_3_-xBaTiO_3_ [9], (1−x)PbZrO_3_-xPbTiO_3_ [10], (Pb_(1−x)_La_x_)(Zr_y_Ti_(1−y)_)_(1−0.25x)_O_3_ [11], (Pb_(1−x)_Ba_x_)(Zr_(1−y)_Ti_y_)O_3_ [12], (Ba_(1−x)_Sr_x_)(Zr_(1−y)_Ti_y_)O_3_ [13], and Pb(Fe_0.5_Nb_0.5_)O_3_ [14].

According to the information outlined hereinabove, it seems reasonable to simultaneously dope BaTiO_3_ ceramics with lanthanum and iron ions. The addition of La^3+^ ions into the Ba^2+^ position reduces the number of covalent bonds. The substitution of Fe^3+^ ions in place of Ti^4+^ ions leads to limitations on the movement of oxygen octahedra [15,16]. The effect thereof may confer a significant possibility to regulate the temperature of the phase transition from the ferroelectric to the paraelectric phase, a substantial increase in the dielectric constant at room temperature (*Tr*), and to obtain the interesting electrical properties of this compound [17,18]. The influence of the lanthanum admixture on the dielectric and electrical properties of BaTiO_3_ ceramics has been described in earlier papers [19,20,21]. The obtained results expressly indicate that doping barium titanate with lanthanum ions, in the amount of 0.4 mol% (Ba_0.996_La_0.004_Ti_0.999_O_3_—BLT4), leads to the creation of a ceramic material with an exceptionally high value of electrical permittivity and interesting electrical properties [22,23,24,25].

Further optimization of the material parameters of the discussed ceramics should lead to an increase in piezoelectric properties, which are particularly desirable from the point of view of sensory applications.

One of the common known methods for improving the properties of ceramic materials is a modification with an admixture of atoms. Various methods of doping BaTiO_3_ and BLT ceramics are widely described in the literature. Modification occurs by substituting homovalent ions (e.g., Sr^2+^) [25,26,27] or heterovalent ions (e.g., Sm^3+^, Er^3+^, Ho^3+^, or La^3+^) [28,29,30,31]. One of the most interesting types of admixture is iron ions. A crucial fact is that Fe (110) and BaTiO_3_ (100) have a perfect match of lattice constants (at the level of 98.6%), which enables the epitaxial growth of multilayered Fe/BaTiO_3_ layer by layer, without unmatched dislocations [32]. Moreover, the addition of Fe^3+^ ions induces magnetic moments in BaTiO_3_, which does not cause the disappearance of the ferroelectric properties of this compound [10]. The presented facts impact the choice of iron ions as a modifier of BLT ceramics. The influence of Fe ions on dielectric and electrical properties has been widely described in our previous paper. The modified materials show a classical phase transformation. Together with an increase in Fe^3+^ ion concentration, a decrease in the values of electric permittivity as well as a Curie constant were proved, and a small shift in the Curie temperature towards lower values occurred. Furthermore, the obtained materials show a slight PTCR effect. In the present paper, we broadly describe the influence of iron dopant on piezoelectric properties of Ba_0.996_La_0.004_Ti_0.999_O_3_ (BLT4) ceramics. The obtained results allow the determination of the most efficient iron ion concentration in terms of piezoelectric properties.

## 2. Experimental Section

The iron-modified Ba_0.996_La_0.004_TiO_3_ ceramics were obtained by conventional solid-state reaction techniques with the thermal sintering of the oxide and carbonate compounds. The appropriate amounts of reagents—BaCO_3_, La_2_O_3_, Fe_2_O_3_, TiO_2_ (SIGMA-ALDRICH, Poznań, Poland)—were derived from stoichiometric calculations on the basis of the general formula Ba_0.996_La_0.004_Ti_1−y_Fe*_y_*O_3_ (BLTF). The concentration of Fe was equal to *y* = 0.1 mol% (BLTF1), *y* = 0.2 mol% (BLTF2), *y* = 0.3 mol% (BLTF3), and *y* = 0.4 mol% (BLTF4). According to this formula, the Fe^3+^ ions substitute the Ti^4+^ ions in the B site of the perovskite structure, which leads to the limitation of the movement of oxygen octahedra [15,16]. Obtained powders were weighed and milled for 8 h in a Fritsch “Pulverisette 5” planetary mill (Fritsch, Idar-Oberstein, Germany), pressed into disc-shaped pellets, and placed in a sealed crucible. The synthesis was conducted at a temperature of 1223 K for 6 h. After the process, samples were crushed, milled, formed again into their final form of 10 mm diameter pellets, and sintered at the temperature of 1523 K for 2 h. The described procedure was repeated twice more, after each sintering. The technological conditions of the second and third sintering processes were equal to 1573 K for 2 h and 1623 K for 2 h, respectively. The goal of multiple sintering processes was to obtain ceramic materials with well-formed microstructure.

A microstructure of the obtained ceramics was checked by means of a scanning electron microscope HITACHI S-4700 (SEM) (Tokyo, Japan) with an energy dispersive *X*-ray spectrometer (EDS and EPMA) by NORAN–Vantage (Middleton, WI, USA), which allowed confirmation of the expected composition of the ceramics. *X*-ray measurements for the obtained composite samples were performed using a diffractometer (PANalytical, Phillips X’Pert Pro, Eindhoven, The Netherlands) (Cu–Kα radiation). The data were collected at room temperature in the 2θ range from 10° to 65°, in steps of 0.02 degrees, with the integration time of 4 s/step.

For other measurements presented in the article, 0.6 mm thick samples were cut and polished. For electrical contact, both flat faces of the samples were coated with silver paste and burned at 923 K for 6 h. Before piezoelectric measurements, the samples were immersed in silicone oil, poled under an external DC electric field of 30 kV/cm applied at 343 K for 15 min, and then slowly cooled in the electric field to room temperature.

The electromechanical coefficients were obtained based on the resonance and antiresonance method [33]. The effective electromechanical *k_eff_* and the mechanical quality factor *Q_M_* were obtained by using the following formulas (independently from the specific conditions of vibration made) [34,35]:

$$k_{eff}^{2}=\frac{f_{a}^{2}-f_{r}^{2}}{f_{a}^{2}}$$

where

$f_{a}$—antiresonance frequency;
$f_{r}$—resonance frequency.

$$Q_{M}=\frac{1}{2\pi f_{s}R_{z}C_{l}\left(1-\frac{f_{r}^{2}}{f_{a}^{2}}\right)}$$

where

$f_{s}$—series resonance frequency;
$R_{z}$—resonance impedance;
*C_l_*—is the capacitance under 1 kHz.

Moreover the relative permittivity along the poling direction ($\varepsilon_{33}^{T}$) was calculated from the [36]:

$$\varepsilon_{33}^{T}=\frac{C_{l}d}{A}$$

where

*A*—is the area of the electrode;
*d*—sample thickness.

In order to calculate *k_eff_* and *Q*_M_, an Agilent E4980A LRC meter (Agilent, Santa Clara, CA, USA) was used to measure capacitance, dielectric loss tangent, resonance frequency, anti-resonance frequency, and impedance of discussed ceramics.

The piezoelectric coefficient *d*_33_ was measured using a Berlincourt-type “quasi-static” meter (Wide-Range d_33_ Tester Meter—APC, Mackeyville, PA, USA).

Electric field-induced polarization was measured by using a Sawyer–Tower circuit based on High Voltage Power Supply Digital Meter BOB 1000DM (KEPCO, New York, NY, USA) and Low Noise Current Preamplifier SR570 (Stanford Research Systems, Sunnyvale, CA, USA). The electric field strength and frequency used in measurements were equal to 30 kV/cm and 50 Hz, respectively.

## 3. Results and Discussion

**Figure 1:**
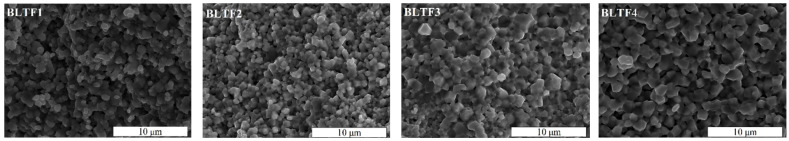
SEM images of BLTF1, BLTF2, BLTF3, and BLTF4 ceramics (5000× magnification) [37].

Figure 1 shows the scanning electron micrographs of Ba_0.996_La_0.004_Ti_1−y_Fe_y_O_3_ ceramics with Fe^3+^ concentrations in the range from 0.1 ≤ y ≤ 0.4 mol%, performed on the fractured surface of the ceramics at room temperature, at 5000× magnification. The surface of the fracture goes along the boundaries between grains, which is typical for material with large, well-shaped, and hard grains. The microstructure of investigated samples shows homogeneous element distribution. Presented images clearly show the densely packed, fine-grained microstructure. The grains are well developed and have an angular shape. The images reveal that the iron admixture caused the increase in average grain size from 0.5 μm for BLTF1 to 2 μm for BLTF4.

An energy dispersive X-ray spectrometer (NORAN–Vantage, Middleton, WI, USA) was used to check the distribution of individual elements within the grains. The EDS investigations show that the obtained ceramics contain only the elements introduced as substrates, with no other impurities detected in the spectrum. The content of barium and titanium oxides slightly differs from the theoretical stoichiometry, whereas the content of lanthanum and iron oxides is encumbered by a large uncertainty, which is related to their small participation in the whole mass of the sample, smaller than the threshold of device detection (Table 1).

**Figure 2:**
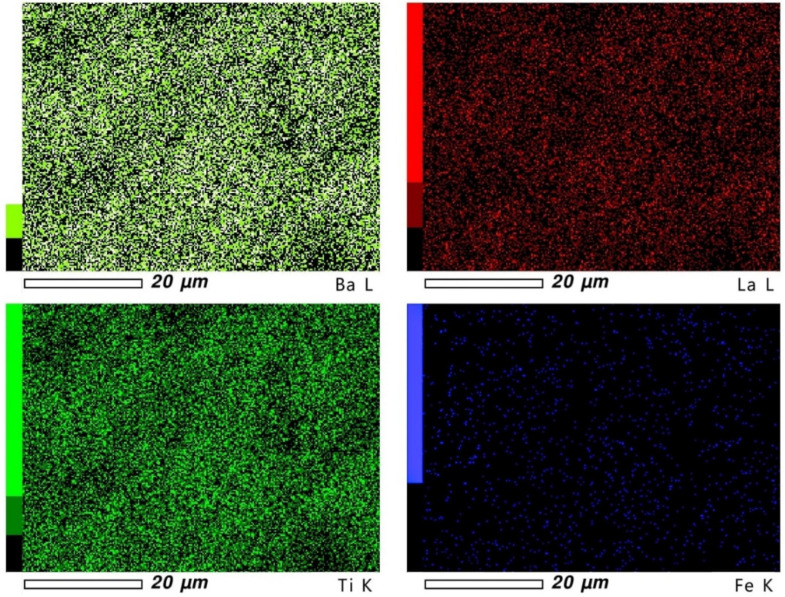
Mapping images for BLTF1 ceramics.

**Figure 3:**
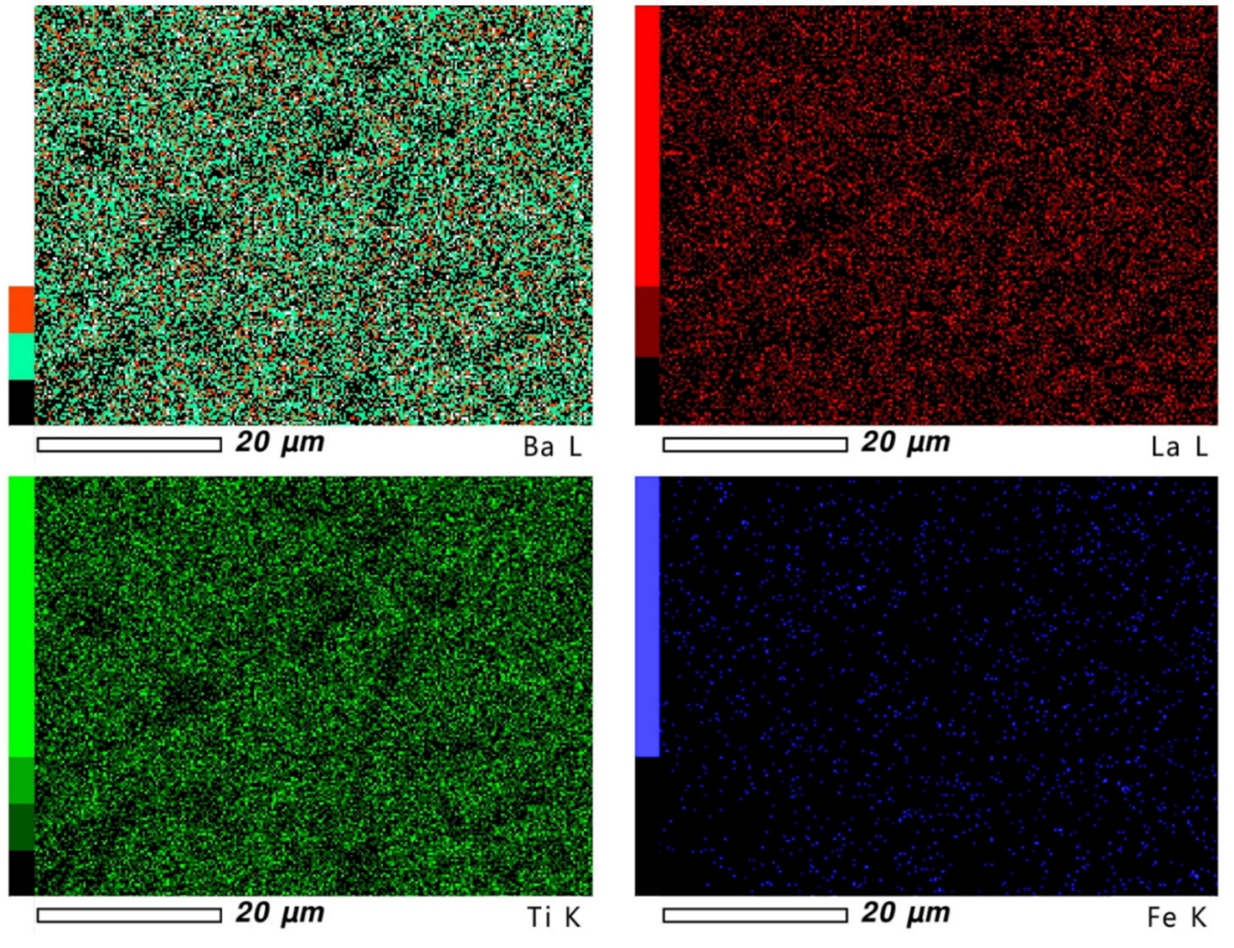
Mapping images for BLTF4 ceramics.

The surfaces of the samples were checked in terms of element distribution homogeneity (Figure 2 and Figure 3). The presence of each element is shown on the mapping in the form of points, where density informs about its concentration. The obtained results indicate that the element distribution is homogeneous in all samples, with some places characterized by lower concentration due to the microstructural features of the material.

**Figure 4:**
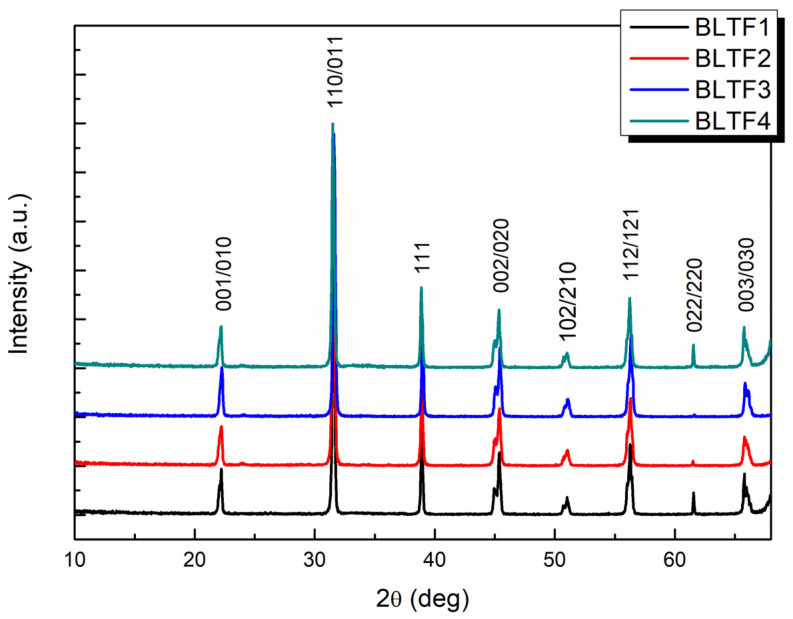
XRD patterns Ba_0.996_La_0.004_Ti_1−y_Fe_y_O_3_ (y = 0.001, 0.002, 0.003, and 0.004).

The phase identification of samples was carried out by XRD (X-ray diffractometer—X’Pert PRO PANalytical, Eindhoven, The Netherlands); the results were detailed in a previous article [37]. Figure 4 illustrates the XRD patterns of the discussed compounds at room temperature and shows that the introduction of iron ions into the crystal lattice does not result in a significant change in peak position. Small deviations in the intensity and the position of diffraction lines can be attributed to the ceramic texturing occurring as a result of the pressing process.

The phase analysis revealed that at room temperature (T_r_ < T_C_) the obtained BLTF ceramic has a single-phase perovskite structure with tetragonal symmetry and the P4mm space group. Using the Rietveld method, on the basis of the obtained X-ray spectra, the unit cell parameters were determined for the BLTF1 ceramics as *a*_0_ = 0.3993 nm, *c*_0_ = 0.4032 nm, for BLTF2 as *a*_0_ = 0.3992 nm, *c*_0_ = 0.4031 nm, for BLTF3 as *a*_0_ = 0.3993 nm, *c*_0_ = 0.4031 nm, and for BLTF4 as *a*_0_ = 0.3993 nm, *c*_0_ = 0.4030 nm. The Fe^3+^ admixture causes a slight reduction in the unit cell volume from *V* = 64.307 × 10^−30^ m^3^ for 0.1 mol% to *V* = 64.269 × 10^−30^ m^3^ for 0.4 mol% of iron, which indicates the correct incorporation of the dopant into the unit cell of the base BLT4 ceramic, ordering its crystal structure, and decreasing the concentration of structural defects [37].

**Figure 5:**
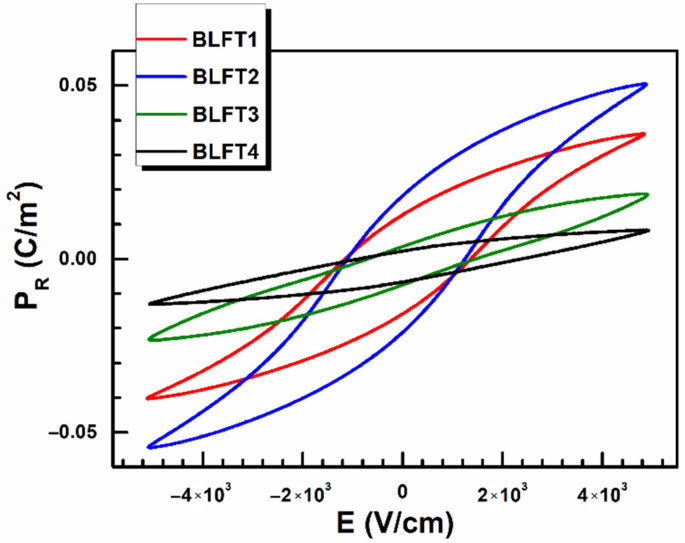
Influence of Fe dopant on hysteresis curve for BLFT1–4.

The influence of iron dopant on the dielectric and electrical properties of BLT4 ceramics was well described in paper [37]. This paper focused on the changes in the ferroelectric and piezoelectric properties of BLT4 ceramics caused by Fe^3+^ ions admixture. A significant influence of the dopant on the re-polarization processes was noted. Figure 5 shows the ferroelectric hysteresis loops of the discussed ceramic materials. The dependences were obtained at room temperature.

**Figure 6:**
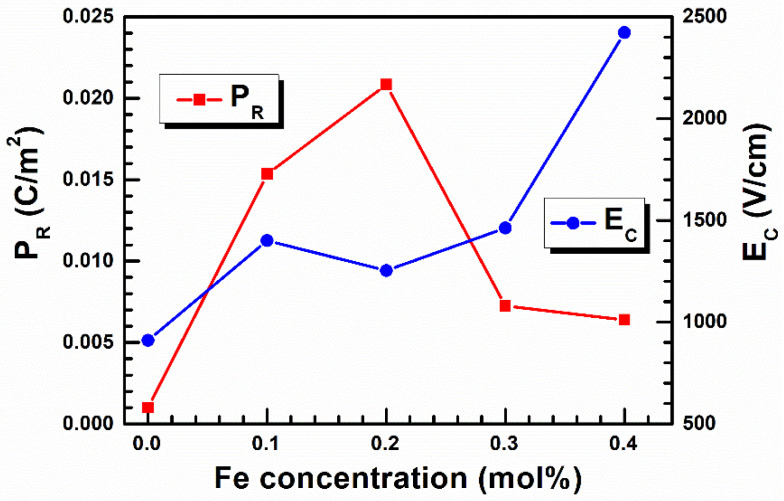
The remnant polarization and coercive field as a function of iron ion concentration.

The *P_R_*(*E*) relationships for samples containing 0.1 and 0.2 mol% have a shape similar to the classic hysteresis loop. Still, a further increase in iron concentration leads to a narrowing of the loop and a decrease in remnant polarization value (Figure 6).

The obtained results indicate that low concentrations of iron admixture enhance the ferroelectric properties, but a further increase in the admixture causes their rapid weakening. The threshold in this process appears to be a concentration between 0.2 and 0.3 mol%. Similar effects were not observed in the case of dielectric properties [37].

**Figure 7:**
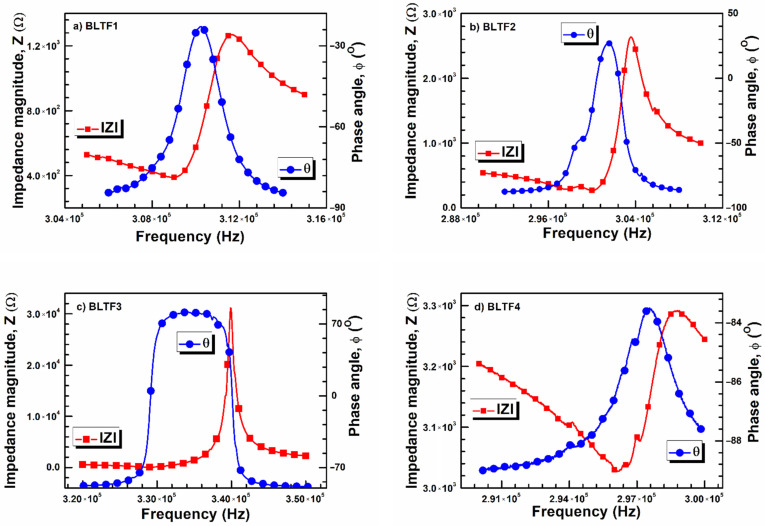
Frequency dependence of impedance magnitude *Z* and phase angle ϕ for BLTF1 (**a**), BLTF2 (**b**), BLTF3 (**c**), and BLTF4 (**d**) ceramics.

In order to determine the influence of iron admixture on the piezoelectric properties of BLT4 ceramics, the impedance spectra of the discussed materials were measured at room temperature. The results recorded at the standard resonance frequency are presented in Figure 7. In the case of the BLT sample containing 0.3 mol% of iron, the results point at the rapid increase in impedance magnitude value (*Z*), which is increased by three orders of magnitude compared to that obtained for other discussed samples. Moreover, the maximum of the phase angle (ϕ) has an immensely broadened character. These facts point to the rapid enhancement of the electromechanical coefficient.

The variation of the calculated values of electromechanical coefficients and dielectric parameters for a frequency of 1 kHz are presented in Table 2.

Table 2 presents the values of the analyzed parameters of the measured BLTF samples: static capacitance along the poling direction ($C_{33}^{T}$), relative permittivity along the poling direction ($\varepsilon_{33}^{T}$), dielectric loss tangent (tan *δ*), effective electro-mechanical coupling coefficient (*k_eff_*), mechanical quality factor (*Q_M_*), and piezoelectric coefficient (*d*_33_).

With the increase in iron content in the discussed ceramics, the static capacity measured along the polarization direction significantly decreases, while the relative electric permittivity increases, reaching a maximum value of $\varepsilon_{33}^{T}$ = 2259 × 10^−12^ for BLT4 ceramics with 0.2 mol% of Fe^3+^. A further increase in the iron content results in a decrease in the value of the discussed electric permittivity. It should be noted that this tendency is linear, and for BLTF4 ceramics, the value of *ε*_33_ is much lower than that of the base material.

The obtained results clearly show that both the dielectric constant and the tangent of the angle of dielectric losses of BLTF ceramics changed significantly under the influence of the impurity, with optimal values achieved for the concentration ranging from 0.2 to 0.3 mol% Fe^3+^. If the iron concentration is increased further, the desired effects are lost. Moreover, the changes in dielectric properties described above are correlated with an increase in the value of the piezoelectric coefficient *d*_33_ and the electromechanical coefficients *k_p_* and *k_eff_* (Table 2). It is noteworthy that the large value of the piezoelectric coefficient equal to *d*_33_ = 159 pC/N recorded for the sample with 0.3 mol% iron; this sample also shows the optimal value of the mechanical quality factor *Q_M_* = 337. As a comparison, the value of the coefficient *d*_33_ in the reference sample BLT4 is eight times smaller and equal to 20 pC/N, while the value of the *Q_M_* coefficient is six times lower (*Q_M_* = 55) [38]. The increase in the Fe^3+^ dopant up to 0.4 mol% causes a decrease in both discussed coefficients. One of the reasons for such improvement in piezoelectric properties is connected with the decrease in grain size of admixture ceramics. The grain size of pure BLT4 ceramics is equal to about 5 μm [39], whereas in modified ceramics it is in the range from 0.5 μm to 2 μm. Grain size plays an essential role in the shaping of ferroelectric and piezoelectric properties [40]. The authors of the cited work proved that in ceramics based on barium titanate, the reduction of the grain size leads to an increase in the value of piezoelectric coefficients. This is connected with the decrease in domain size in the case of materials with small grains. In such materials, participation of the grain boundary and domain wall in the volume of sample is significant. Consequently, they became “soft” and sensitive, resulting in the increase in piezoelectric properties [41]. The piezoelectric mechanism is complex, and the discussed behavior could also influence the structural defects and their periodicity, and the movement of mentioned ferroelectric domains walls [42].

The recorded value of the *d*_33_ coefficient is large, but about four times lower than the extreme values recorded for the system of lead-free ceramics (1−x)Ba(Zr_0.20_Ti_0.80_)O_3_-x(Ba_0.70_Ca_0.30_)TiO_3_. For a solid solution with x = 50, the coefficient *d*_33_ is equal to 620 pC/N [43]. Another example of a material characterized by a huge value of the *d*_33_ parameter is the ceramic 0.96(K_0.5_Na_0.5_)_0.95_Li_0.05_Nb_(1−x)_Sb_x_O_3_-0.04BaZrO_3_(KNLNS_x_-BZ). The *d*_33_ coefficient in that case equals 425 pC/N [44].

**Figure 8:**
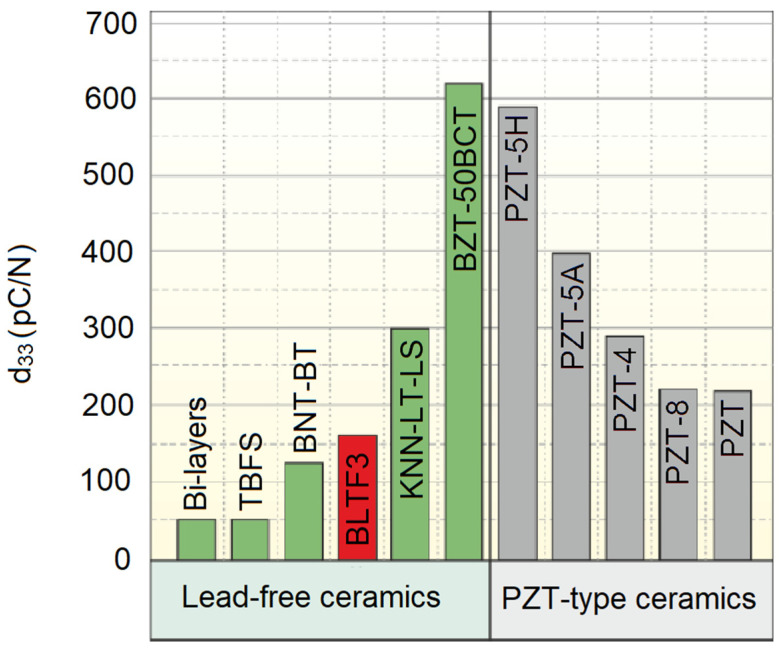
*d*_33_ values at room temperature for BLTF3 ceramics compared to other piezoelectric materials. This figure is adapted from [43].

Figure 8 depicts a comparison of the BLTF3 ceramics with other high-performing piezoelectric materials, in which lead-containing materials are the leaders, i.e., the entire family of PZT ceramics (soft (H), hard (A), modified and undoped), as well as lead-free ceramics: layered perovskite Aurivillius structure (bi-layered), tetragonal tungsten bronze ceramic (TBSF), ceramic (Bi_0.5_Na_0.5_)TiO_3_-BaTiO_3_ (BNT-BT), ceramic (K, Na, Li) (Nb, Ta, Sb) O_3_ (KNN-LT-LS), and ceramics (1−x)Ba(Zr_0.2_Ti_0.8_)O_3_-x(Ba_0.7_Ca_0.3_)TiO_3_ (BZT-50BCT) [43].

It should also be emphasized that with the increase in iron ion content in BLTF ceramics, there is a clear tendency to increase in the values of the effective electromechanical coupling coefficient (*k_eff_*), as well as the mechanical quality factor (*Q_M_*) summarized in Table 2. This trend can be attributed to increased compressive stresses induced by iron ions with a smaller radius than barium ions in the BLTF unit cell [45].

The discussed ceramics are the base for the further improvement of piezoelectric properties. One of the most promising ways to the goal is the introduction of additional co-dopants into A sublattice. The results described in the paper [46] indicate that it is a more effective method for enhancing piezoelectric modulus *d*_33_ and increasing residual polarization than dopants modification of B sublattice. The other way to high piezoelectric activity is the modification of microstructure by employing the different processing techniques (for example, templated grain growth) to achieve high-density ceramics characterized by oriented, well-developed grains [47].

## 4. Conclusions

Pure and iron ion-modified BLT4 ceramics were prepared by the solid state reaction method. The XRD measurements show that the obtained materials are characterized by a single-phase crystal structure with tetragonal symmetry corresponding to the P4mm space group. The research confirmed the correct selection of technological conditions. The mechanical quality factor *Q_M_* and the piezoelectric coefficient *d*_33_ for pure BLT4 ceramics are equal to 55 and 20 pC/N, respectively [38]. The presented results reveal that a small amount of iron dopant significantly improved the piezoelectric properties (Table 2). However, if the modifier exceeds the determined threshold value of about 0.3 mol%, the parameters describing the mentioned properties start to deteriorate. It is worth noting that excellent piezoelectric properties are characteristic of ceramics containing 0.3 mol% of admixture, mainly a high piezoelectric parameter *d*_33_ = 159 pC/N, making BLTF ceramics a competitor to other lead-free materials. This composition is the alternative for the materials commonly used for sensors and actuator applications. Moreover, the ceramic material could be applied as transducers in ultrasonic imaging and parking sensors [48].

## Figures and Tables

**Table 1:** Theoretical and experimental contents of elements (calculation for simple oxides) for BLTF ceramics.

Formula	Content of Oxides from EDS (wgt.%) (Measurement)	Theoretical Content of Oxides (wgt.%)	Accuracy (wgt.%)
BLTF1 ceramics			
BaO	64.95	65.48	0.53
La_2_O_3_	0.30	0.28	0.02
TiO_2_	34.64	34.21	0.43
Fe_2_O_3_	0.11	0.03	0.08
BLTF2 ceramics			
BaO	65.54	65.48	0.06
La_2_O_3_	0.00	0.28	0.28
TiO_2_	34.30	34.18	0.12
Fe_2_O_3_	0.16	0.07	0.09
BLTF3 ceramics			
BaO	65.11	65.48	0.37
La_2_O_3_	0.83	0.28	0.55
TiO_2_	33.90	34.14	0.24
Fe_2_O_3_	0.15	0.10	0.05
BLTF4 ceramics			
BaO	65.59	65.48	0.11
La_2_O_3_	0.31	0.28	0.03
TiO_2_	33.81	34.11	0.30
Fe_2_O_3_	0.29	0.14	0.15

**Table 2:** The dielectric and piezoelectric parameters for the Fe^3+^-modified BLT4 samples.

Parameters	BLTF1	BLTF2	BLTF3	BLTF4
$C_{33}^{T}$ (pF)	849	846	459	190
$\varepsilon_{33}^{T}$·10^−12^	1762	2259	1481	708
tan *δ*	0.0198	0.0151	0.0110	0.0312
*k_eff_*	0.135	0.157	0.249	0.131
*Q_M_*	88	118	337	55
*d*_33_ (pC/N)	70	102	159	40

## Data Availability

Not applicable.
